# Supplementary material for: Convergent Epigenetic Evolution Drives Relapse in Acute Myeloid Leukemia
Source: bioRxiv. 2023 Oct 10:2023.10.10.561642. Preprint. [Version 1] doi: 10.1101/2023.10.10.561642 (PMC10592718; doi:10.1101/2023.10.10.561642)
Supplement: Supplement 1 [file NIHPP2023.10.10.561642v1-supplement-1.pdf]

bioRxiv preprint doi: https://doi.org/10.1101/2023.10.10.561642; this version posted October 10, 2023. The copyright holder for this preprint (which was not certified by peer review) is the author/funder, who has granted bioRxiv a license to display the preprint in perpetuity. It is made available under aCC-BY 4.0 International license.

Supplementary data associated with *Convergent Epigenetic Evolution Drives Relapse in Acute Myeloid Leukemia*

Kevin A. Nuno[1,2,3,4,13], Armon Azizi[2,3,4,5,13], Thomas Köhnke[2,3,4], Caleb A. Lareau[6,7], Asiri Ediwirickrema[1,2,3,4], M. Ryan Corces[1,2,3,4,8,9,10], Ansuman T. Satpathy[6,7,11,12], Ravindra Majeti[2,3,4*]

Affiliations

1. Cancer Biology Graduate Program, Stanford University School of Medicine, Stanford, CA, USA
2. Institute for Stem Cell Biology and Regenerative Medicine, Stanford University School of Medicine, Stanford, CA, USA
3. Cancer Institute, Stanford University School of Medicine, Stanford, CA, USA
4. Department of Medicine, Division of Hematology, Stanford University School of Medicine, Stanford, CA, USA
5. University of California Irvine School of Medicine, Irvine, California
6. Department of Pathology, Stanford University, Stanford, CA, USA
7. Program in Immunology, Stanford University, Stanford, CA, USA
8. Gladstone Institute of Neurological Disease, San Francisco, California.
9. Gladstone Institute of Data Science and Biotechnology, San Francisco, California.
10. Department of Neurology, University of California San Francisco, San Francisco, California
11. Parker Institute for Cancer Immunotherapy, Stanford University, Stanford, CA, USA
12. Gladstone-UCSF Institute of Genomic Immunology, San Francisco, CA, USA
13. These authors contributed to this work equally

*corresponding author; email: rmajeti@stanford.edu

bioRxiv preprint doi: https://doi.org/10.1101/2023.10.10.561642; this version posted October 10, 2023. The copyright holder for this preprint (which was not certified by peer review) is the author/funder, who has granted bioRxiv a license to display the preprint in perpetuity. It is made available under aCC-BY 4.0 International license.

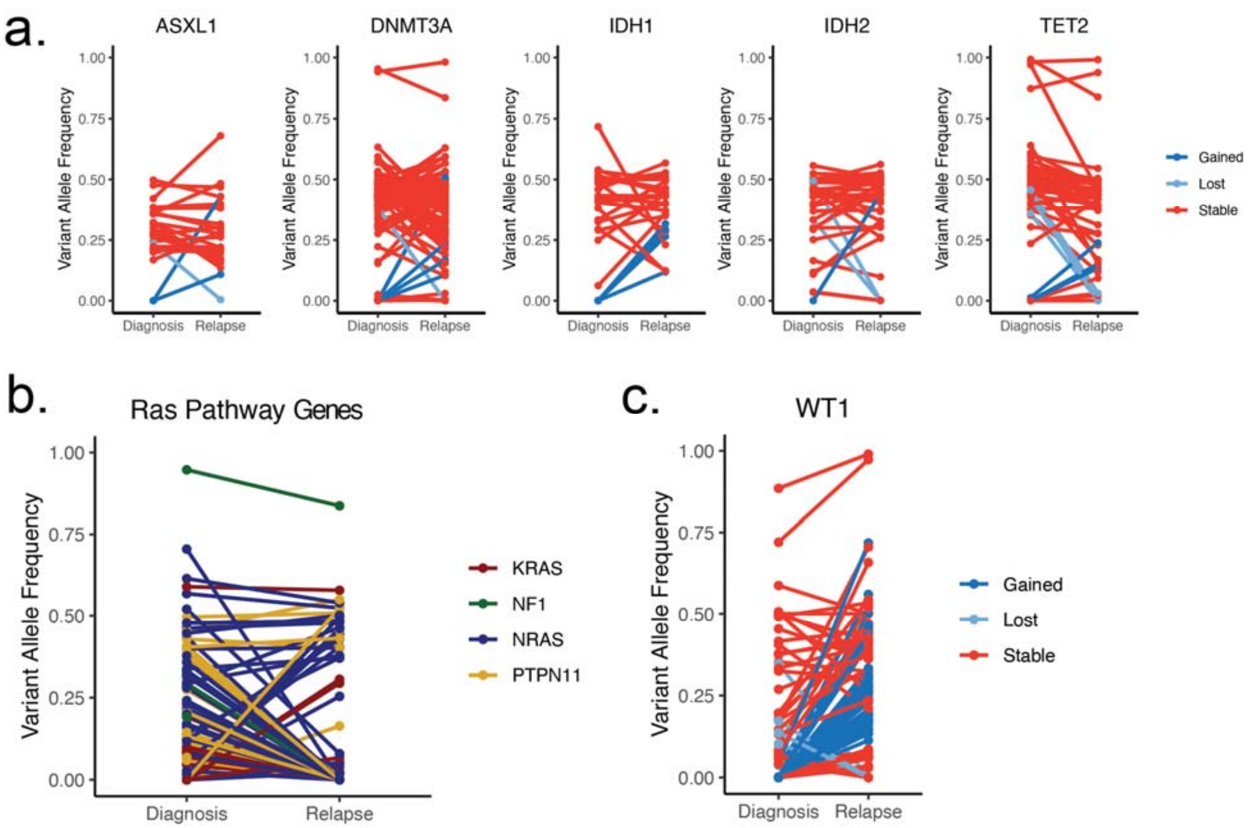

Supplementary Figure 1: Plots depicting change in variant allele frequency for detected "pre-leukemic" epigenetic modifier mutations (a), RAS pathway mutations (b), or WT1 mutations (c) in meta-analysis relapsed AML cohort. Lines connect samples from the same patient between disease timepoints, color coded according to mutation dynamic (red = stable, blue = gained at relapse, yellow = lost at relapse) (a, c) or according to RAS family member (b).

bioRxiv preprint doi: https://doi.org/10.1101/2023.10.10.561642; this version posted October 10, 2023. The copyright holder for this preprint (which was not certified by peer review) is the author/funder, who has granted bioRxiv a license to display the preprint in perpetuity. It is made available under aCC-BY 4.0 International license.

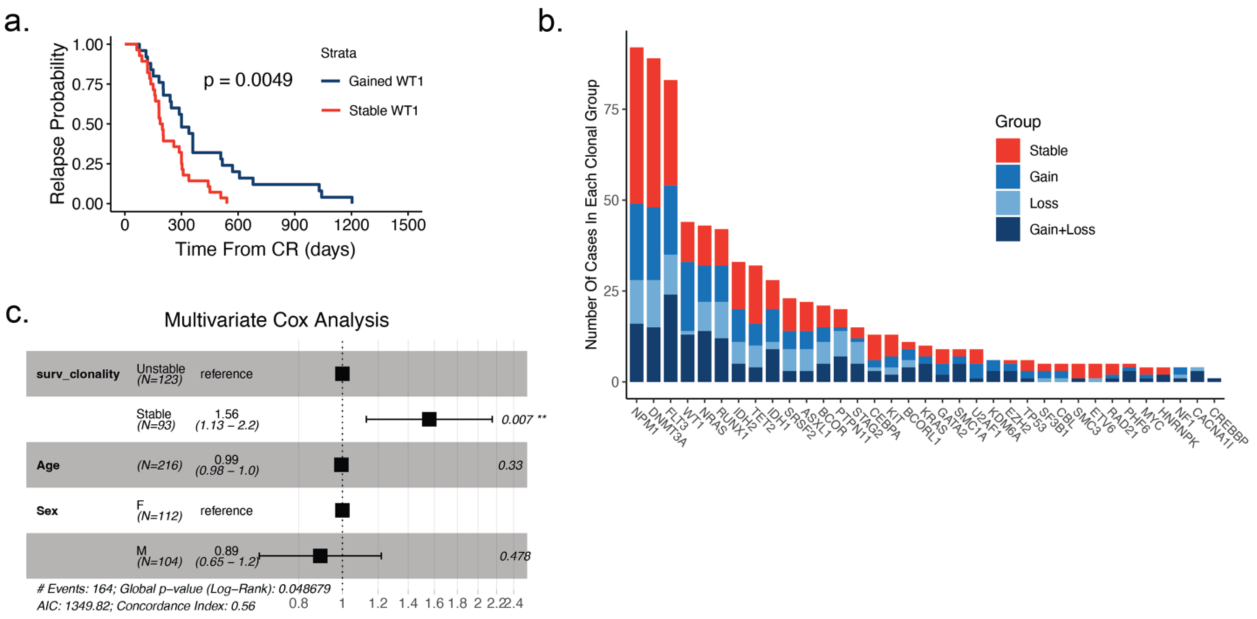

<u>Supplementary Figure 2</u>: a.) Time to relapse analysis of meta-analysis cohort patients according to WT1 mutation status between diagnosis and relapse (blue = gained WT1 mutation at relapse, red = WT1 mutation stable at relapse). b.) Plot depicting recurrent mutations of meta-analysis cohort AML patients. Fraction of each bar graph depicts the proportion of mutants that fall in the designated clonal group colored according to key shown at right. c.) Multivariate Cox analysis of survival data from meta-analysis patient cohort data accounting for mutation clonality, age, and sex.

bioRxiv preprint doi: https://doi.org/10.1101/2023.10.10.561642; this version posted October 10, 2023. The copyright holder for this preprint (which was not certified by peer review) is the author/funder, who has granted bioRxiv a license to display the preprint in perpetuity. It is made available under aCC-BY 4.0 International license.

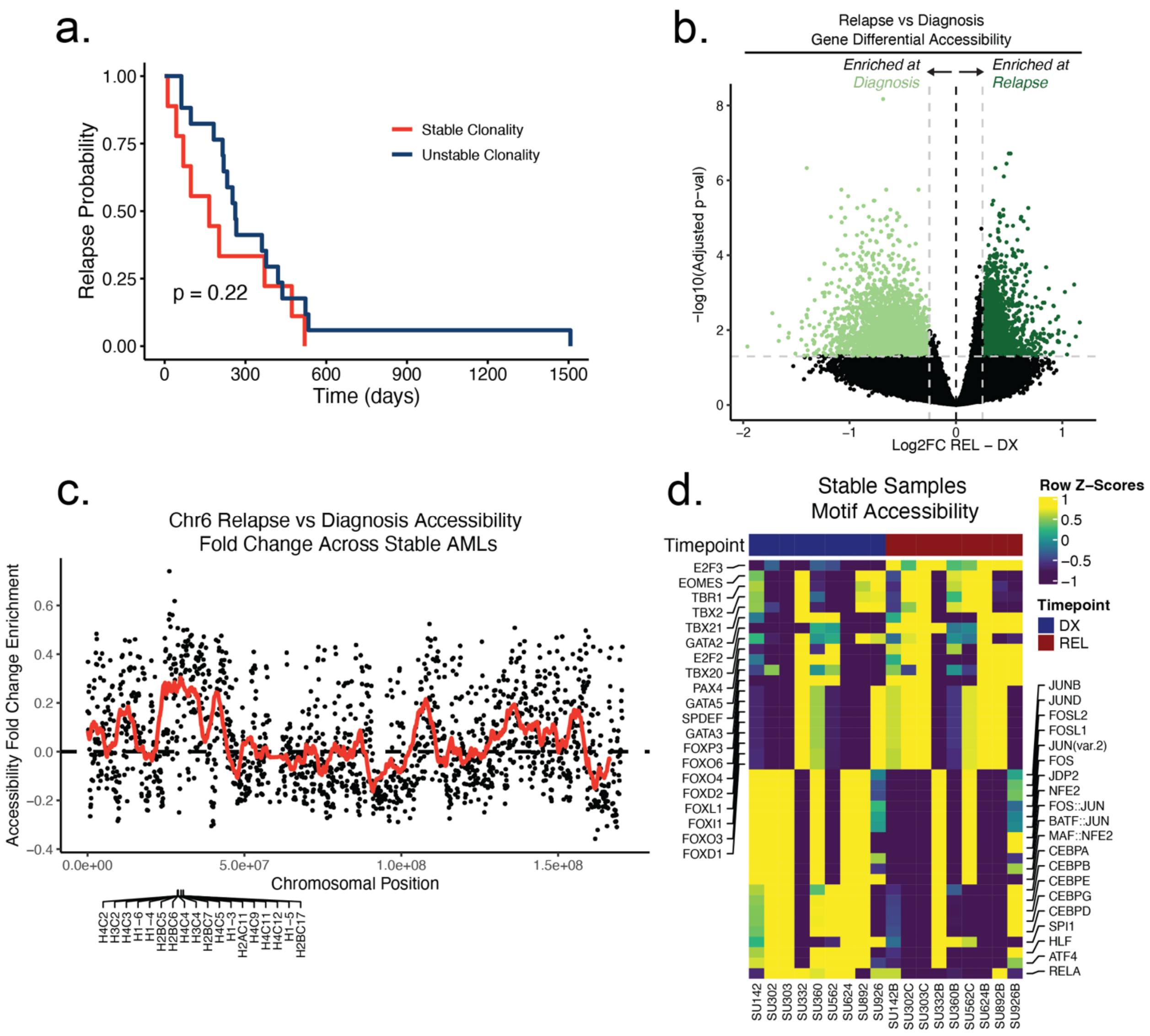

Supplementary Figure 3: a.) Time to relapse analysis of Stanford cohort patients according to clonality assessed by driver mutation genotyping. b.) Volcano plot of differential accessibility between diagnosis and relapse across all ATAC-seq peaks in genomically stable samples. Peaks considered significantly up or down-regulated if adjusted p value < 0.05 and absolute log2 fold change > 0.25. c.) Plot depicting fold change in accessibility of tiled 100kb bins on chromosome 6 across stable samples at relapse. Dots indicate single bin fold change at relapse and red line shows the running fold change average across bins. Genes that were found to be significantly differential in accessibility are labeled at the bottom of the plot. d.) Heatmap of motif

bioRxiv preprint doi: https://doi.org/10.1101/2023.10.10.561642; this version posted October 10, 2023. The copyright holder for this preprint (which was not certified by peer review) is the author/funder, who has granted bioRxiv a license to display the preprint in perpetuity. It is made available under aCC-BY 4.0 International license.

accessibility at diagnosis and relapse for genomically stable samples. Only the top 40 differentially accessible motifs are shown.

bioRxiv preprint doi: https://doi.org/10.1101/2023.10.10.561642; this version posted October 10, 2023. The copyright holder for this preprint (which was not certified by peer review) is the author/funder, who has granted bioRxiv a license to display the preprint in perpetuity. It is made available under aCC-BY 4.0 International license.

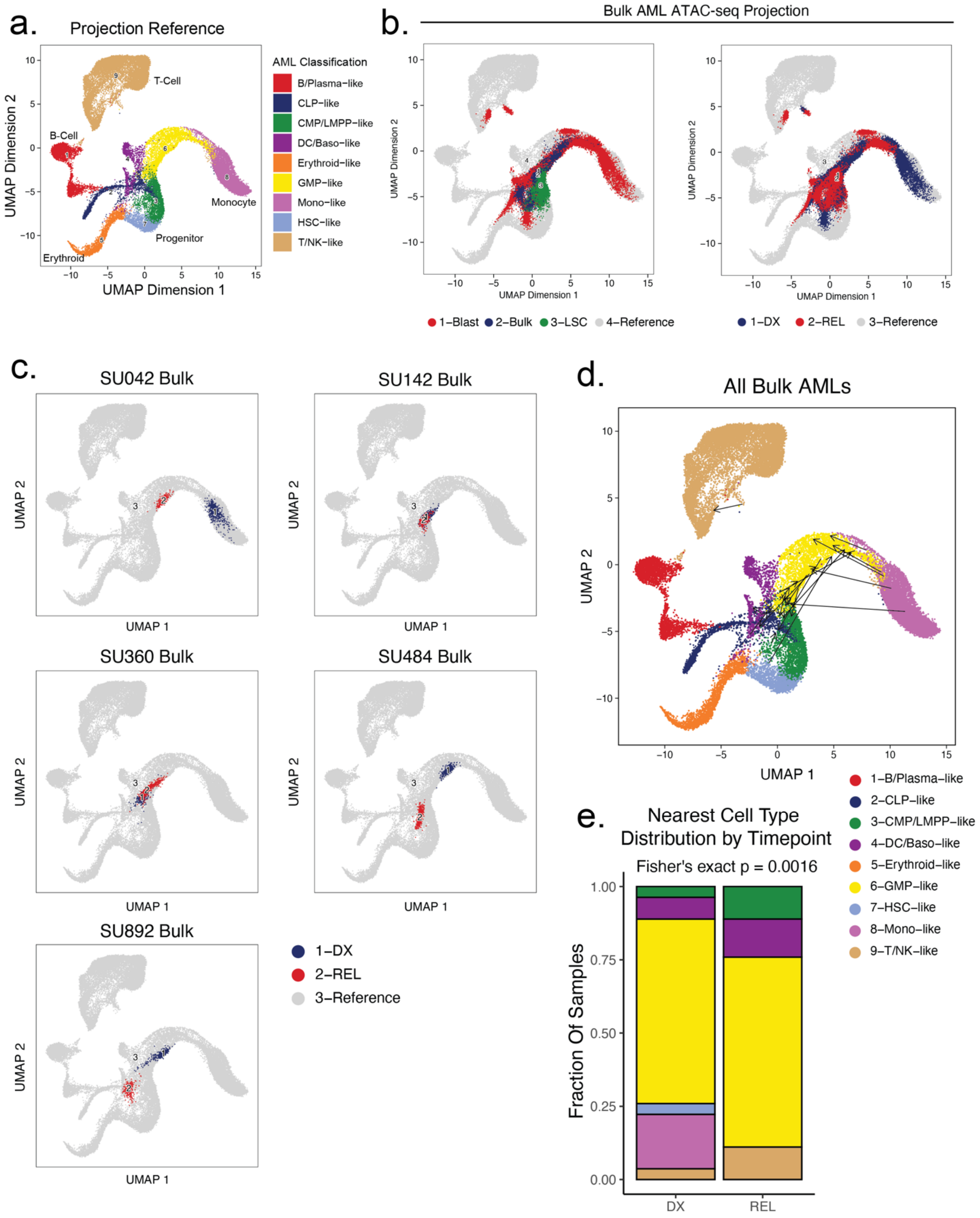

Supplementary Figure 4: a). Healthy cell reference manifold projection of single-cell ATAC-seq data from Granja et al *Nat. Biotech* 2019. Healthy hematopoietic cell categories color coded

bioRxiv preprint doi: https://doi.org/10.1101/2023.10.10.561642; this version posted October 10, 2023. The copyright holder for this preprint (which was not certified by peer review) is the author/funder, who has granted bioRxiv a license to display the preprint in perpetuity. It is made available under aCC-BY 4.0 International license.

according to key shown below. b) Bulk AML ATAC-seq data “pseudo”-single cell profiles projected to healthy hematopoietic reference umap. Projected cells are colored either by sorted cell population (left) or time of acquisition (right). c) Projection of bulk AMLs from 2 patients using pseudo-single cell approach. Diagnosis samples shown in blue, relapse samples shown in red. d) Plot depicting the change in projection between diagnosis and relapse across all samples. Arrows connect the average projection location of diagnosis cells to the average projection location of relapse cells for each patient. e) Distribution of the closest mapped cell type across all AML Blast (non-LSC enriched) samples. Diagnosis and relapse distributions were compared using a fisher’s exact test.

bioRxiv preprint doi: https://doi.org/10.1101/2023.10.10.561642; this version posted October 10, 2023. The copyright holder for this preprint (which was not certified by peer review) is the author/funder, who has granted bioRxiv a license to display the preprint in perpetuity. It is made available under aCC-BY 4.0 International license.

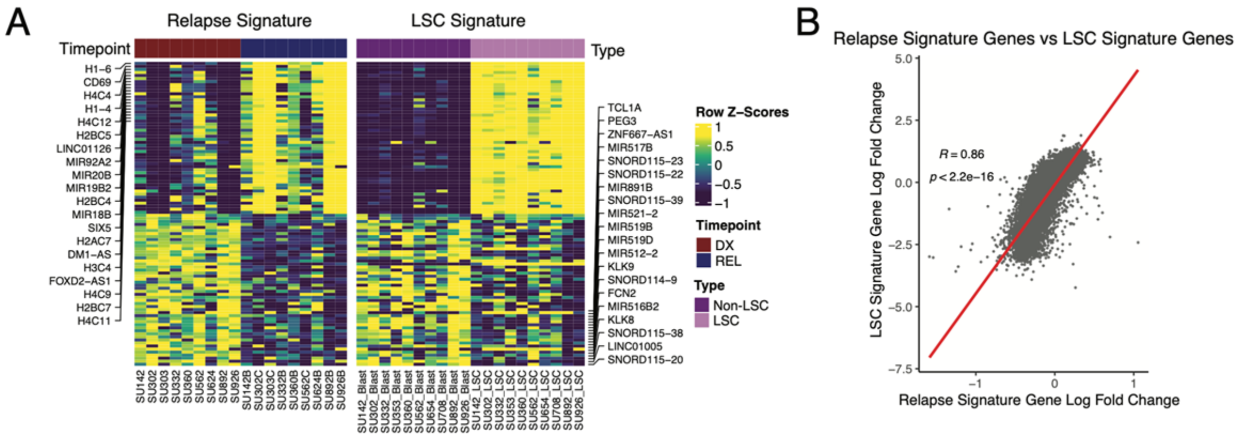

<u>Supplementary Figure 5:</u> Comparison between AML relapse signature and LSC signature. Relapse signatures were derived by comparing relapse timepoints to diagnosis timepoints in stable AML patients. LSC signatures were derived by comparing LSCs to non-LSC populations at diagnosis across patients with sortable LSC populations. Relapse chromatin changes were compared to LSC chromatin changes using gene accessibility scores. A) Heatmap of the most differential gene accessibility scores in relapse samples at diagnosis and relapse timepoints (left), and LSC vs Non-LSC populations (right). B) Dot-plot depicting the correlation between relapse signature gene accessibility score fold changes and LSC signature gene accessibility score fold changes.

bioRxiv preprint doi: https://doi.org/10.1101/2023.10.10.561642; this version posted October 10, 2023. The copyright holder for this preprint (which was not certified by peer review) is the author/funder, who has granted bioRxiv a license to display the preprint in perpetuity. It is made available under aCC-BY 4.0 International license.

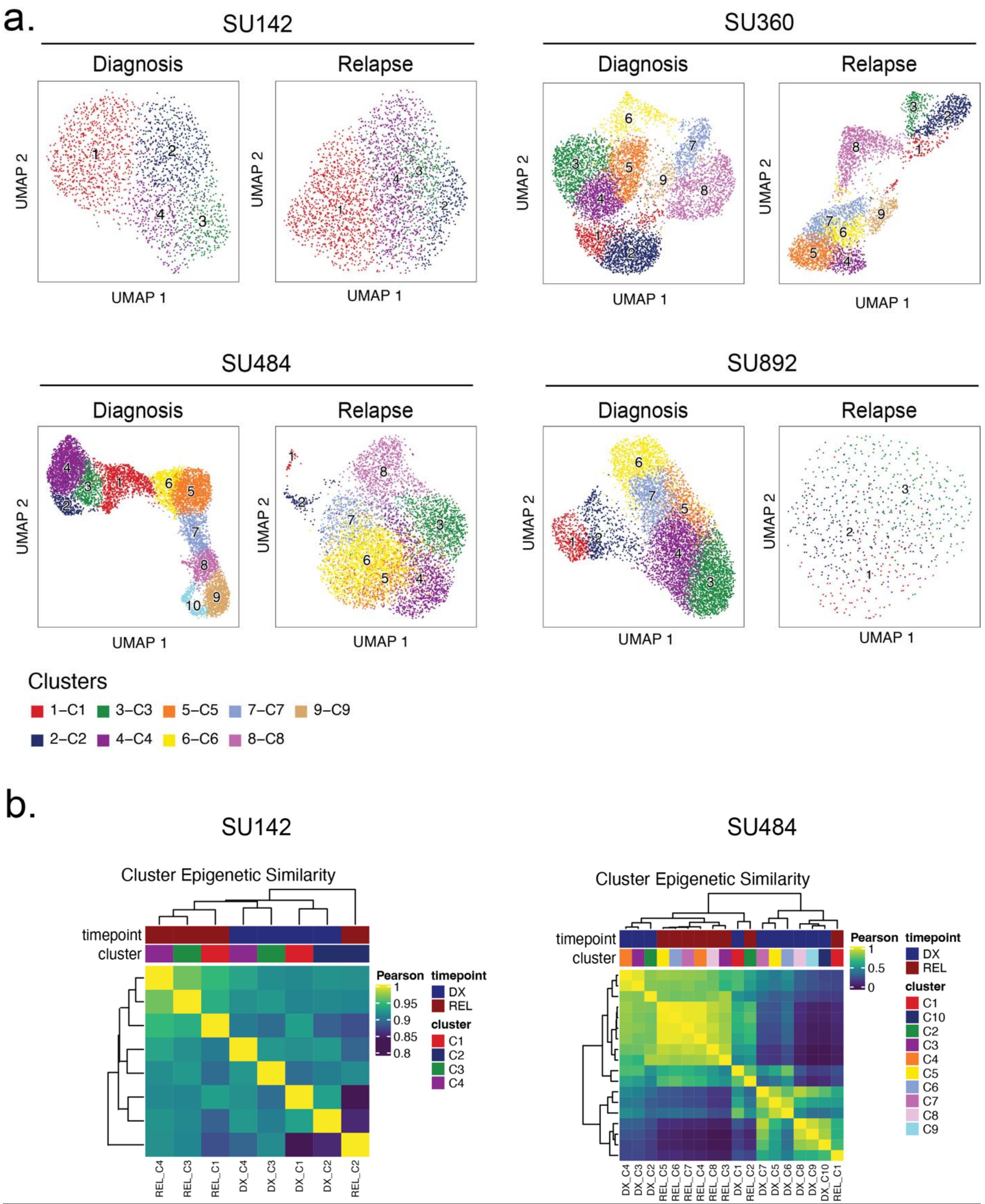

Supplementary Figure 6: a) UMAP plots of clustering analyses performed for the four patient samples indicated (diagnosis sample at left, relapse at right). b) Hierarchical clustering analysis

bioRxiv preprint doi: https://doi.org/10.1101/2023.10.10.561642; this version posted October 10, 2023. The copyright holder for this preprint (which was not certified by peer review) is the author/funder, who has granted bioRxiv a license to display the preprint in perpetuity. It is made available under aCC-BY 4.0 International license.

of scATAC-seq data from diagnosis and relapse cell clusters from patients SU142 (left) and SU484 (right).

bioRxiv preprint doi: https://doi.org/10.1101/2023.10.10.561642; this version posted October 10, 2023. The copyright holder for this preprint (which was not certified by peer review) is the author/funder, who has granted bioRxiv a license to display the preprint in perpetuity. It is made available under aCC-BY 4.0 International license.

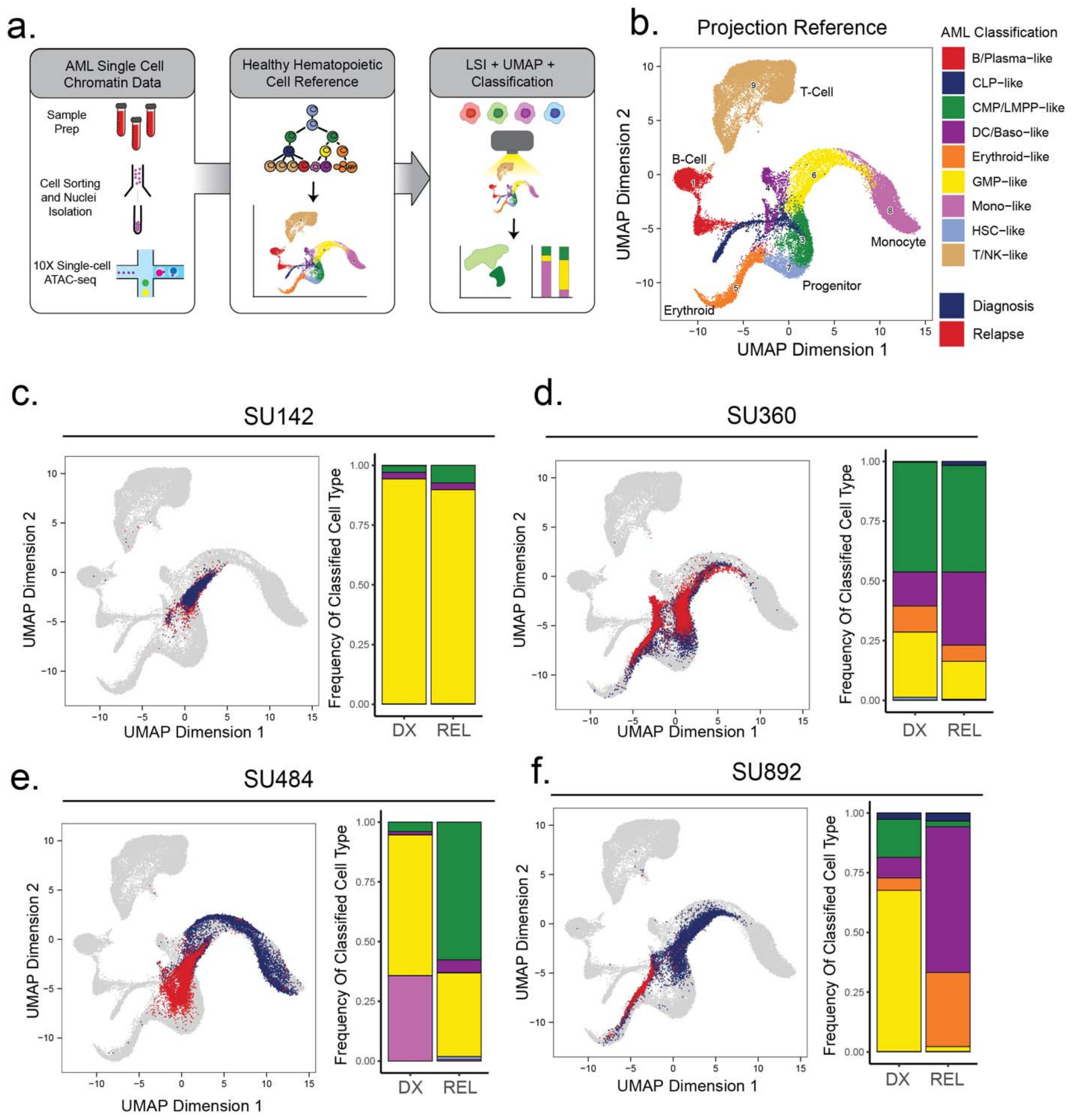

Supplementary Figure 7: a) Schematic of strategy for healthy cell LSI projection analysis of single-cell ATAC-seq diagnosis/relapse data b) Healthy cell reference of single-cell ATAC-seq data from Granja et al Nat. Biotech 2019. Healthy hematopoietic cell categories color coded according to key shown at bottom. c-f) UMAP projections of single-cell ATAC-seq samples onto healthy cell reference manifolds for indicated patients. Barplot of nearest cell type classification fractions across all cells at diagnosis and relapse is shown for each patient.

bioRxiv preprint doi: https://doi.org/10.1101/2023.10.10.561642; this version posted October 10, 2023. The copyright holder for this preprint (which was not certified by peer review) is the author/funder, who has granted bioRxiv a license to display the preprint in perpetuity. It is made available under aCC-BY 4.0 International license.

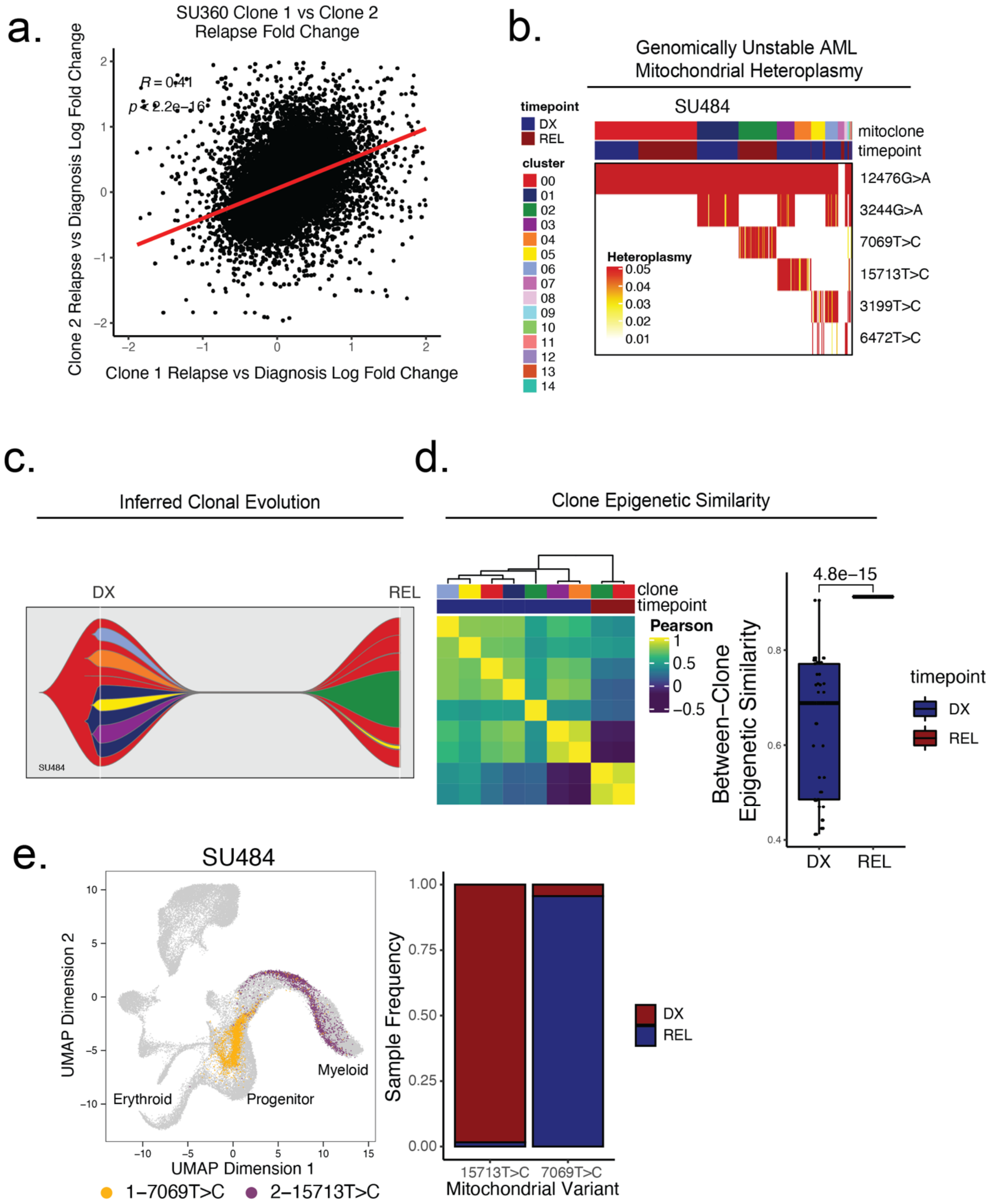

bioRxiv preprint doi: https://doi.org/10.1101/2023.10.10.561642; this version posted October 10, 2023. The copyright holder for this preprint (which was not certified by peer review) is the author/funder, who has granted bioRxiv a license to display the preprint in perpetuity. It is made available under aCC-BY 4.0 International license.

Supplementary Figure 8: a) Comparison of relapse vs diagnosis accessibility fold change across all peaks between mitoclone 1 and mitoclone 2 in SU360 (R=0.41, p<2.2e-16). b) Heatmap of mitochondrial variant heteroplasmy values across all single cells for SU484. Only variants passing filtering criteria are shown. Cells are ordered based on the cluster they are assigned to. C) Fishplot of inferred mitochondrial clone evolution at diagnosis and relapse based on mitochondrial cluster frequencies in (B). D) Heatmap of epigenetic similarity across all major mitoclones (left). Boxplot of inter-clone epigenetic similarity at diagnosis and relapse (means compared using unpaired student’s t-test) (right). e) LSI projection of cells containing either 7069T>C or 15713T>C mitochondrial variants to the healthy hematopoietic reference umap (left). Barplot depicting frequency of cells from diagnosis or relapse timepoints containing either 7069T>C or 15713T>C variants (right).

bioRxiv preprint doi: https://doi.org/10.1101/2023.10.10.561642; this version posted October 10, 2023. The copyright holder for this preprint (which was not certified by peer review) is the author/funder, who has granted bioRxiv a license to display the preprint in perpetuity. It is made available under aCC-BY 4.0 International license.

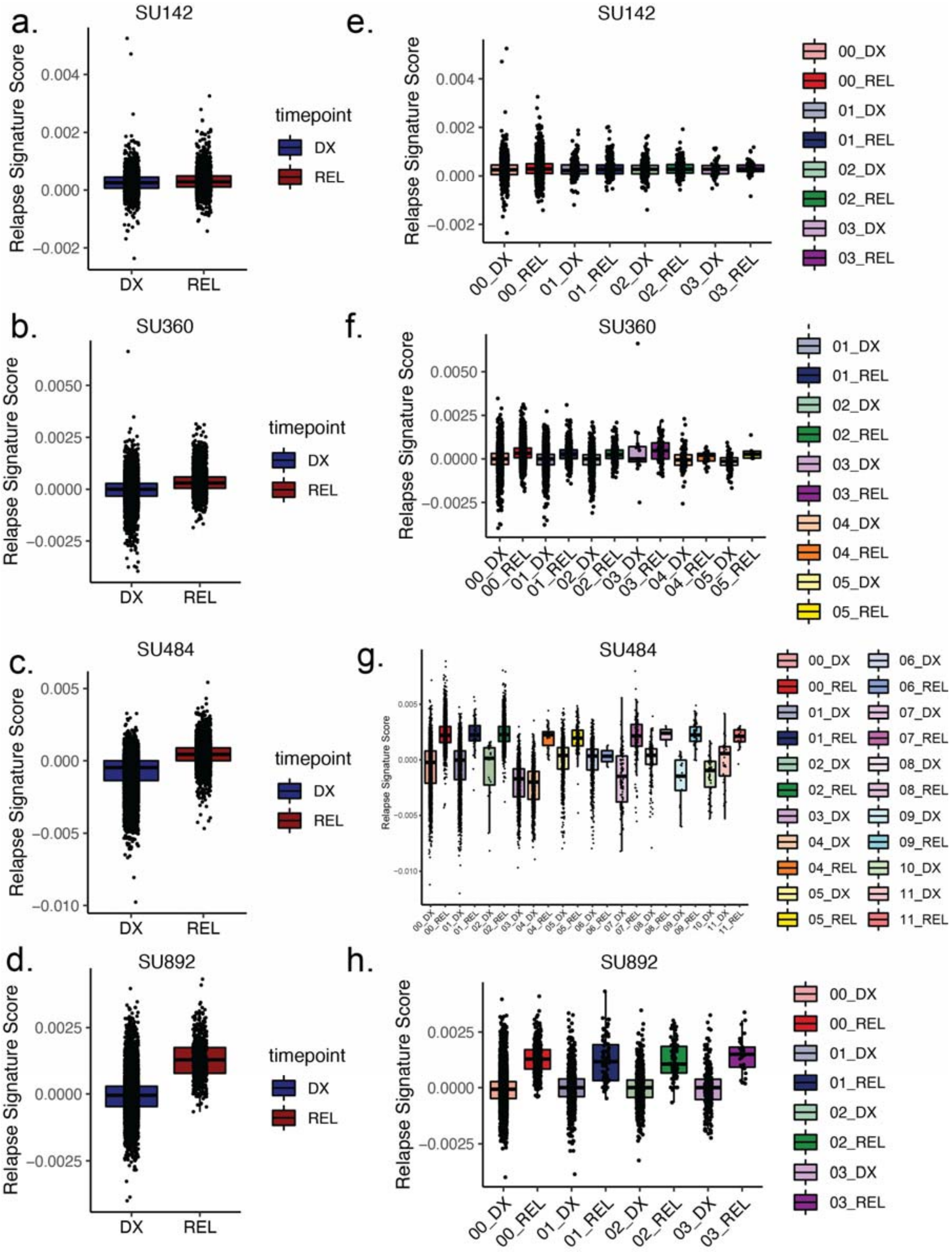

Supplementary Figure 9: a-d) Box plots depicting relapse signature ATAC-seq score calculated for single cells for the indicated patients according to timepoint, diagnosis (left)

bioRxiv preprint doi: https://doi.org/10.1101/2023.10.10.561642; this version posted October 10, 2023. The copyright holder for this preprint (which was not certified by peer review) is the author/funder, who has granted bioRxiv a license to display the preprint in perpetuity. It is made available under aCC-BY 4.0 International license.

shown in blue, relapse (right) shown in red. e-h) Box plots depicting relapse signature score calculated for single cells sorted according to mitoclone designation using mtscATAC-seq for indicated patients. Clones are defined and colored according to the keys shown at right for both diagnosis and relapse samples.
